# Supplementary figures and images for: Exploring the impact of antibody-dependent cellular phagocytosis-related genes on the prognosis of metastatic melanoma
Source: PLoS One. 2025 Oct 9;20(10):e0333916. doi: 10.1371/journal.pone.0333916 (PMC12510546; doi:10.1371/journal.pone.0333916)

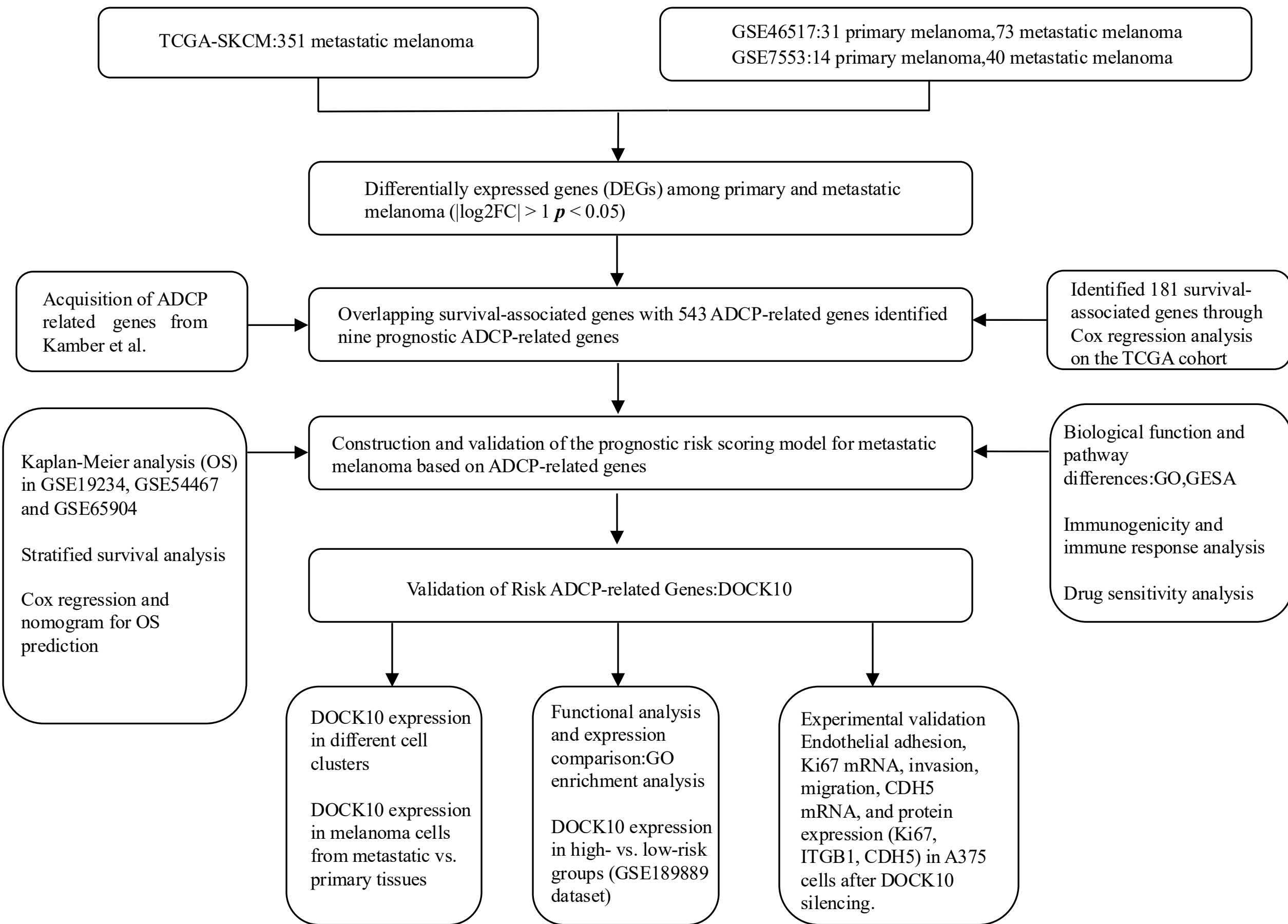

Supplement: S1 Fig — The flowchart illustrates the systematic approach used in this research, including: (1) Data acquisition from TCGA and GEO databases; (2) Identification of differentially expressed genes between primary and metastatic melanoma; (3) Intersection with ADCP-related genes; (4) Feature selection using LASSO regression; (5) Construction of the six-gene prognostic model; (6) Validation in multiple cohorts; (7) Functional analyses including pathway enrichment, immune cell infiltration, and drug sensitivity prediction; (8) Experimental validation of DOCK10 through siRNA knockdown and functional assays. TCGA, The Cancer Genome Atlas; GEO, Gene Expression Omnibus; DEGs, Differentially Expressed Genes; ADCP, Antibody-Dependent Cellular Phagocytosis; LASSO, Least Absolute Shrinkage and Selection Operator; GO, Gene Ontology; GSEA, Gene Set Enrichment Analysis. (PDF) [file pone.0333916.s001.pdf]

**A**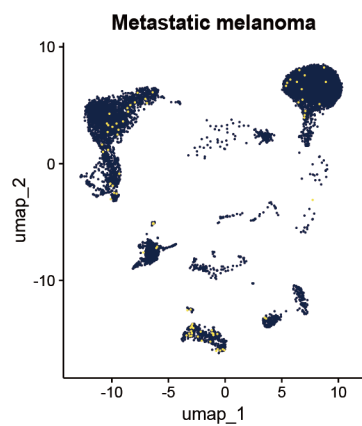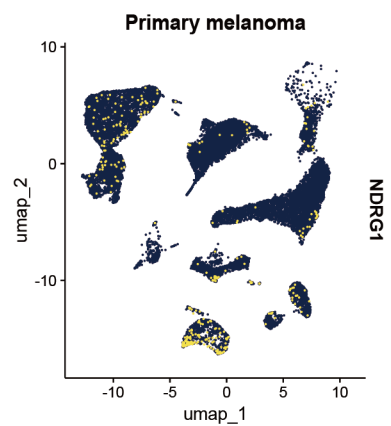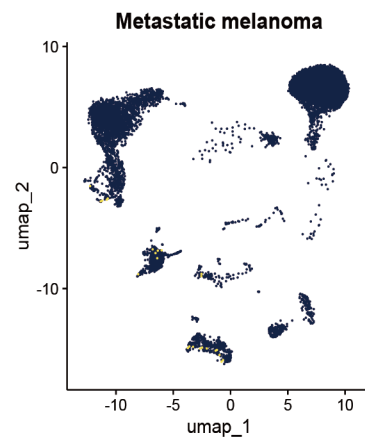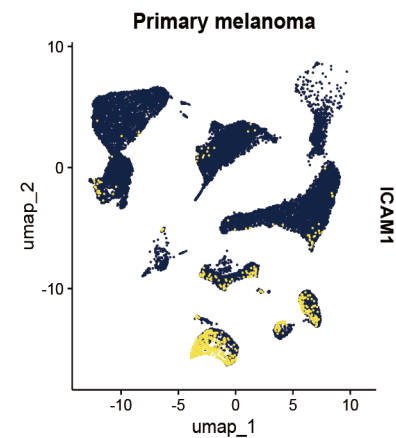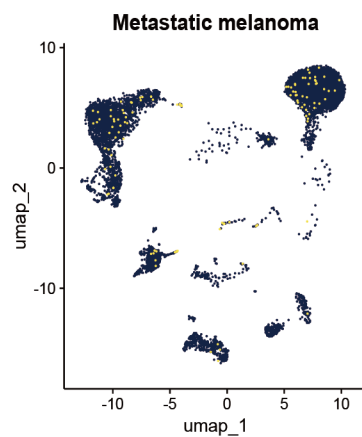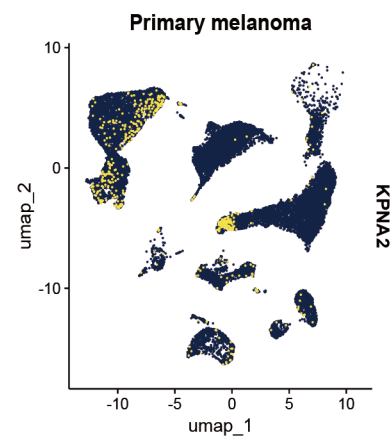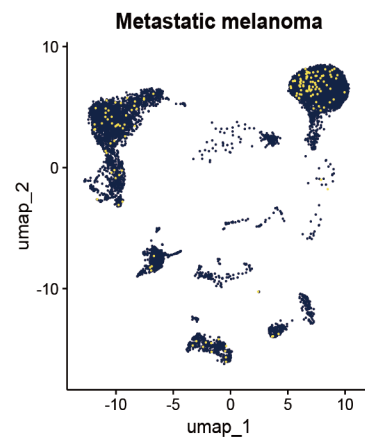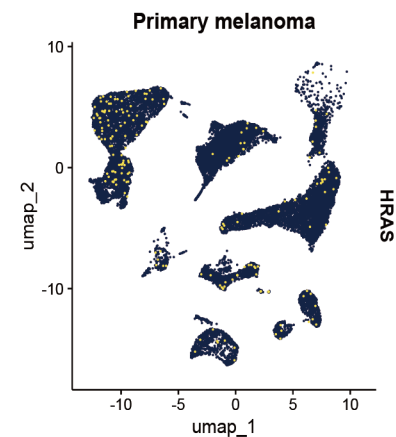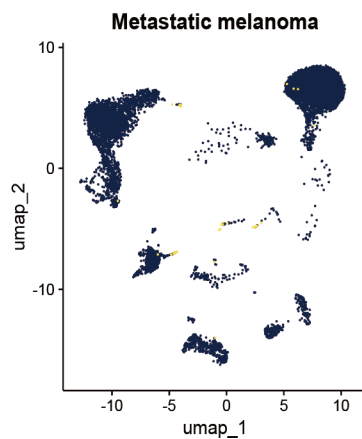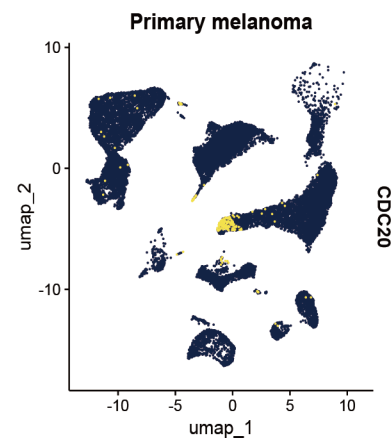

Supplement: S3 Fig — (PDF) [file pone.0333916.s003.pdf]

Raw western blot images in figure8

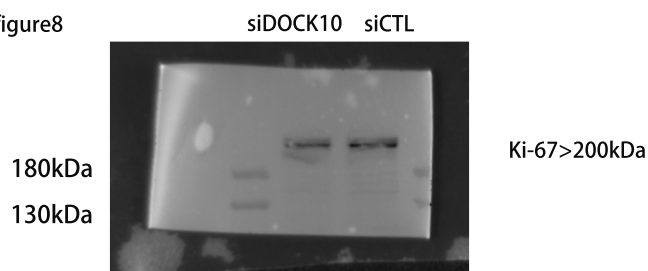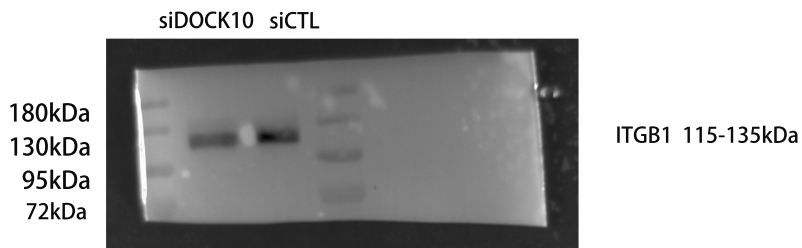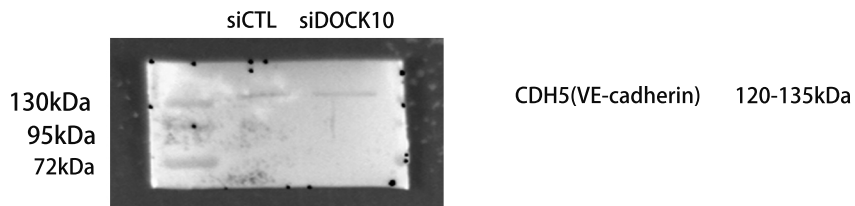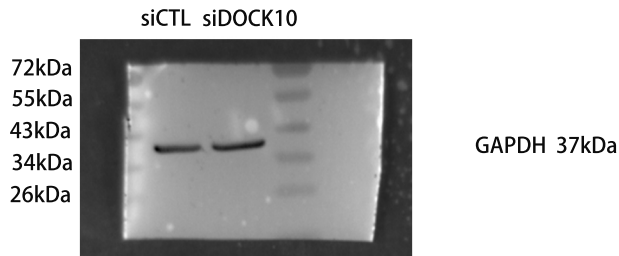

Supplement: S4 Fig — (PDF) [file pone.0333916.s004.pdf]
