# Supplementary material for: Exploring the impact of antibody-dependent cellular phagocytosis-related genes on the prognosis of metastatic melanoma
Source: PLoS One. 2025 Oct 9;20(10):e0333916. doi: 10.1371/journal.pone.0333916 (PMC12510546; doi:10.1371/journal.pone.0333916)
Supplement: S2 Fig — (PDF) [file pone.0333916.s002.pdf]

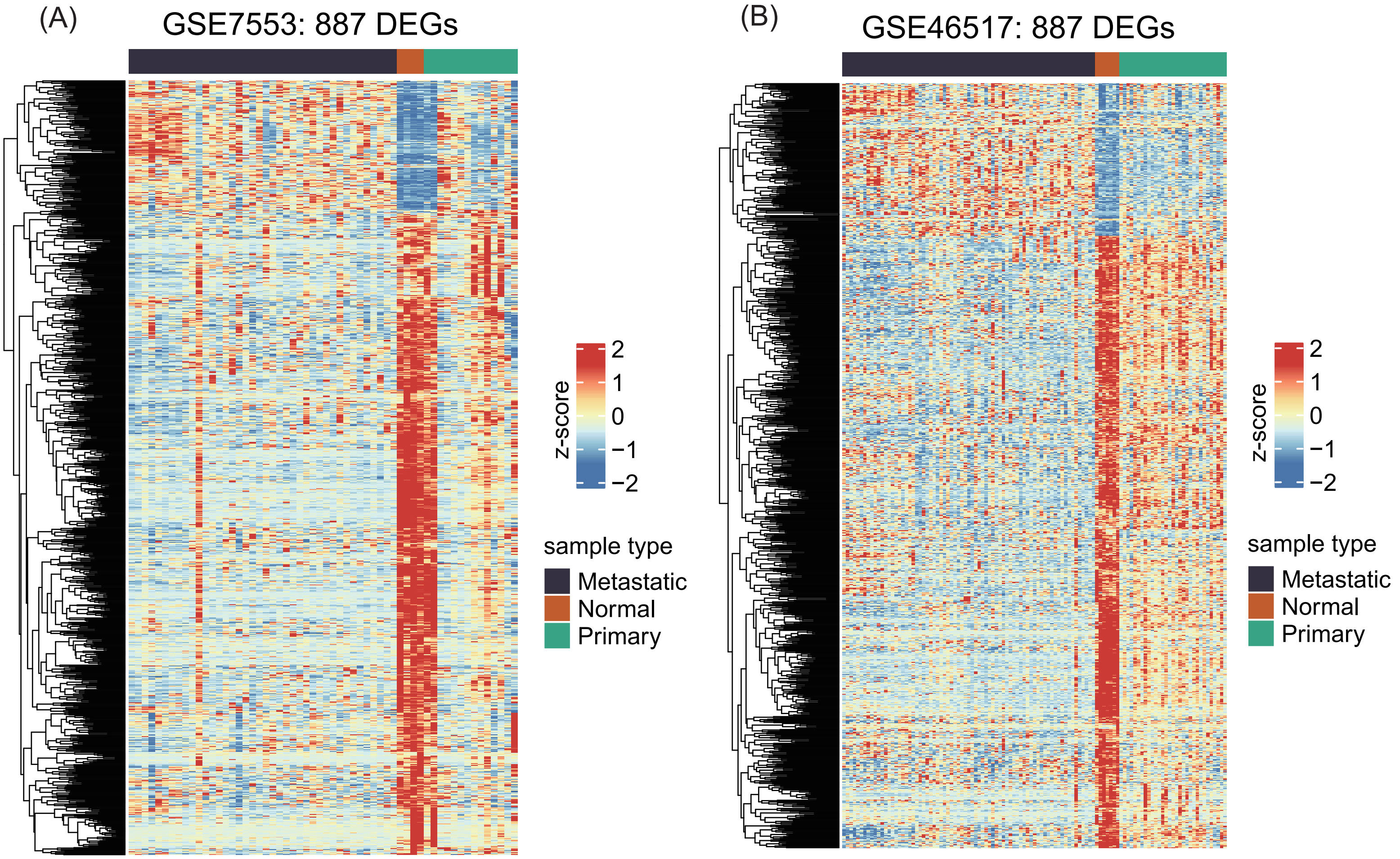

(A)
GSE7553: 887 DEGs
z-score
2
1
0
−1
−2
sample type
Metastatic
Normal
Primary
(B)
GSE46517: 887 DEGs
z-score
2
1
0
−1
−2
sample type
Metastatic
Normal
Primary
