# Supplementary material for: Exploring the impact of antibody-dependent cellular phagocytosis-related genes on the prognosis of metastatic melanoma
Source: PLoS One. 2025 Oct 9;20(10):e0333916. doi: 10.1371/journal.pone.0333916 (PMC12510546; doi:10.1371/journal.pone.0333916)
Supplement: Attachment — Submitted filename: Response to Reviewers.docx [file pone.0333916.s006.docx]

# Response to Reviewers

We would like to thank you for your valuable comments and suggestions on our manuscript "Exploring the impact of Antibody-Dependent Cellular Phagocytosis-Related Genes on the prognosis of metastatic melanoma." Those comments are all valuable and very helpful for revising and improving our paper. We have carefully addressed all the comments and revised our manuscript accordingly, which we hope to meet your approval. Revised portion are red-marked in our manuscript.Please find the revised version, which we would like to submit for your kind consideration.

Looking forward to hear from you.
Thank you and best regards.
Yours sincerely

## Reviewer #1:

**1.Materials & Methods: Data Acquisition: (Page 5, line 108): Please provide any identifier for TCGA datasets (such as barcode, or project name)**

**Response:** We thank the reviewer for this suggestion. We have added the project identifier for the TCGA dataset in the Methods section as follows: "The data used in this study were obtained from TCGA (The Cancer Genome Atlas) database (https://portal.gdc.cancer.gov/) under the project identifier TCGA-SKCM and GEO (Gene Expression Omnibus) database (http://www.ncbi.nlm.nih.gov/geo)."

2.Was there any data normalization performed or any methodology performed to address batch effects?

**Response:** Thank you for highlighting this important aspect. We have added the following statement to the Methods section to clarify our data processing approach: "For TCGA cohorts, quantile normalization was conducted via the 'limma' package in R software. GEO datasets underwent ComBat correction using the sva package for batch effect removal. All expression values were log2-transformed pre-analysis for normality assurance.."

3.Construction of Prognostic Risk Model: (Page 6, line 129): It is unclear what is the target variable that was used to build Cox Regression Analysis.

**Response:** We appreciate this comment. We have clarified this point in the manuscript: "To identify potential prognostic risk genes for metastatic melanoma in the TCGA training set, univariate Cox regression analysis was performed using overall survival (OS) data, which included survival time (in months) and survival status (alive/dead) as the target variables."

4.Construction of Prognostic Risk Model: (Page 7, line 136): What (dependent) variable in the dataset was used as risk-score. We cannot build risk score model with just gene expression information.

**Response:** Thank you for this important point. We have revised this section to clarify: "The risk score was not a pre-existing variable in the dataset but was calculated based on the multivariate Cox regression coefficients and gene expression values. The following algorithm was used to determine each patient's risk score for metastatic melanoma based on their gene expression profiles: Risk score = Σni= βi x Ei where Ei represented the gene expression value, and βi was the regression coefficient."

5.Performance Evaluation: (Page 7: 142): Is there any reference available to choose median risk score as a threshold (cut-off) to categorize as high-risk and low-risk.

**Response:** We appreciate this question. We have included the following rationale for employing the median risk score as a threshold: "In accordance with common practices in cancer prognostic research (Liu et al., 2021; Zhou et al., 2018), patients in the TCGA training set, TCGA validation set, along with GSE19234, GSE54467, and GSE65904 validation cohorts, were categorised as high-risk or low-risk using the median risk score calculated from the TCGA training set as the threshold. This approach ensures balanced group sizes and has been utilized in comparable prognostic model studies."

6.Figure 1: Author can include ADCP related genes to show how 9 genes were identified.

**Response:** We thank the reviewer for this suggestion. We have updated Figure 1 to better illustrate the identification process of the 9 ADCP-related prognostic genes, showing the overlap between differentially expressed genes from GSE46517 and GSE7553 datasets with the ADCP-related genes.

7.Figure 5A, 5B: Could you please explain how do you correlate up-regulated gene with High Risk, and low-regulated gene with Low Risk. Could you please explain how do you correlate NES < 0 with Low Risk and NES > 0 with Low Risk category.

**Response:** Thank you for raising this important point. We have added the following explanation to the "Enrichment Analysis of Differentially Expressed Genes in High- and Low-Risk Categories" section: "In Figure 5, the 'limma' package was employed with |log2FC| > 1 and adjusted P < 0.05 as thresholds to identify differentially expressed genes between high-risk and low-risk groups. Genes showing higher expression in the high-risk group (positive log2FC) were deemed 'up-regulated in high-risk' and linked to a worse prognosis. Conversely, those with higher expression in the low-risk group (negative log2FC) were regarded as 'down-regulated in high-risk' and associated with a better prognosis. Regarding GSEA analysis, the normalized enrichment score (NES) reveals the pathway enrichment direction. Positive NES values signify pathways enriched in the high-risk phenotype, whereas negative NES values denote pathways enriched in the low-risk phenotype. GSVA pathways with a p-value < 0.05 were considered significantly enriched (Liu et al. 2022)."

## Reviewer #2:

1.Authors are requested to check the use of "fc" fragment crystallizable (Fc) in the sentenced indicated below. Page 4, line 84-85. "Specifically, macrophages recognize the crystal fragment (Fc) fragment of antibodies through Fcγ receptors (FcγR), activating phagocytosis to eliminate antibody-tagged tumor cells (Wang et al. 2024)."

**Response:** Thank you for pointing out this error. We have corrected the sentence as follows: "Specifically, macrophages recognize the fragment crystallizable (Fc) portion of antibodies through Fcγ receptors (FcγR), activating phagocytosis to eliminate antibody-tagged tumor cells (Wang et al. 2024)."

2.The phrase 'gotten' in the sentence 'Datas used in this study were gotten from TCGA...' is not appropriate for scientific writing. Additionally, 'datas' should be corrected to 'data' as 'data' is already a plural noun. A more suitable revision would be: 'The data used in this study were obtained from the TCGA (The Cancer Genome Atlas) database and the GEO (Gene Expression Omnibus) database.' Consider using 'obtained' instead of 'gotten' to maintain academic tone and clarity. (Page 5, Line 106-108).

**Response:** We appreciate this correction. We have revised the sentence as suggested: "The data used in this study were obtained from TCGA (The Cancer Genome Atlas) database (https://portal.gdc.cancer.gov/) under the project identifier TCGA-SKCM and GEO (Gene Expression Omnibus) database (http://www.ncbi.nlm.nih.gov/geo)."

3.In materials and methods section: "A total of 543 ADCP-related genes with P < 0.05 were identified." This sentence may not provide enough detail for the readers or the materials and methods section. Authors are requested to clarify or provide more details in this part. (Page 6, Line 122).

**Response:** Thank you for this suggestion. We have expanded this section to provide more detail: "ADCP-related genes were obtained from a study by Roarke A. Kamber et al. (Kamber et al. 2021). In this research, a genome-wide CRISPR knockout and overexpression screen was used in cancer cells and macrophages to systematically identify factors that impede ADCP without bias. A total of 543 ADCP-related genes with P < 0.05 were identified. These genes are involved in various cellular processes including cell surface antigen presentation, Fc receptor signaling, phagocytic machinery, and cytoskeletal reorganization."

4.Page 6, Line 122-124: "ADCP-related genes overlapping with prognosis-related genes in metastatic melanoma were then selected to obtain ADCP-related genes with prognostic significance (ARGs)." Explanation of this sentence was given in the next title (Identification of ADCP-Related Genes) in the materials and methods section. Authors are requested to check and arrange this part.

**Response:** We thank the reviewer for pointing this out. We have reorganized this section for better clarity by moving the explanation to the appropriate section and expanding on the methodology: "To identify ADCP-related genes with potential prognostic value in metastatic melanoma, we performed univariate Cox regression analysis on the TCGA cohort and identified 181 genes significantly associated with survival (p < 0.05). By overlapping these survival-associated genes with 543 ADCP-related genes, we identified nine ADCP-related genes with prognostic significance (ARGs): NDRG1, HRAS, KPNA2, CCNB1, ICAM1, KIF4A, DOCK10, CDC20, and BLM."

5.Page 7, Line 142-144: In the sentence, 'Based on the median risk score in the training set, individuals in the TCGA training set, TCGA cohort, and GSE19234, GSE54467, and GSE65904 validation cohorts were categorised as high-risk or low-risk,' the term 'training set' should be replaced with 'TCGA validation set' to avoid confusion, as it appears to be referring to the validation cohort rather than the training cohort. Please consider revising this for clarity.

**Response:** We appreciate this correction. We have revised the sentence for clarity: "Based on the median risk score calculated from the TCGA training set as the threshold, patients in the TCGA training set, TCGA validation set, and GSE19234, GSE54467, and GSE65904 validation cohorts were categorised as high-risk or low-risk."

6.There is a grammatical error in the title 'Enrichment Analysis of Differentially Expressed Genes in High- and Low-Risk Categories.' The correct plural form of 'category' is 'categories.' Please revise it. Again, on page 8, line 171,175: "among the two categories."

**Response:** Thank you for pointing out this grammatical error. We have corrected the title to "Enrichment Analysis of Differentially Expressed Genes in High- and Low-Risk Categories" and have also corrected the phrase "among the two categories" throughout the manuscript.

7.The authors are encouraged to visualize the 887 common differentially expressed genes (DEGs) identified between metastatic and non-metastatic samples using a heatmap. This could provide a clearer, more intuitive representation of gene expression patterns across the two datasets, highlighting the similarities and differences in expression levels. Heatmaps are an effective way to present large-scale gene expression data and could enhance the interpretability of the results.

**Response:** Thank you for this valuable suggestion. While space limitations prevent us from showing all 887 genes in a heatmap, we have added the following text to provide more context about these DEGs: "The 887 common DEGs identified between metastatic and primary melanoma samples showed distinctive expression patterns that clearly separated the two melanoma types. Although the manuscript's space limitations prevent us from showing all 887 genes, the most significantly dysregulated genes were enriched in pathways related to cell cycle regulation, extracellular matrix remodeling, and immune response. These expression patterns highlight the molecular changes that occur during melanoma progression from primary to metastatic disease."

8.The authors are requested to construct the risk score model using the entire TCGA dataset instead of dividing it into a 6:4 ratio for training and validation. Since three independent external datasets are already utilized for validation, using the full TCGA dataset for model development would improve the robustness of the prognostic risk score. A larger sample size in model training ensures more reliable coefficient estimation and strengthens the overall model performance. The authors should clarify the reasoning behind their current dataset partitioning strategy and discuss its potential impact.

**Response:** We sincerely appreciate this incisive suggestion. In response, we have conducted a comprehensive reanalysis by utilizing the entire TCGA dataset (n=351) for constructing the risk score model. This approach significantly enhanced the statistical power for coefficient estimation, thereby bolstering the overall robustness of the model. Notably, upon comparing the results obtained from using the entire dataset with our original 6:4 ratio training and validation strategy, the model coefficients remained highly stable. This consistency not only validates the reliability of our six-gene signature but also attests to the robustness of our initial dataset partitioning method. Moreover, regarding our original decision to partition the TCGA dataset into a 6:4 ratio for training and validation, this was primarily to ensure an unbiased evaluation of the model's generalization ability within the TCGA cohort itself. By having a separate validation set, we could rigorously test the model's performance on data that it had not encountered during the training phase, which helped in early identification of potential overfitting issues. Although the availability of three independent external datasets for validation provides strong external validation, the internal 6:4 split offered an additional layer of confidence in the model's performance across different subsets of the TCGA data. We acknowledge that using the full TCGA dataset for model development has its merits, and our supplementary analysis demonstrates that our model can withstand this alternative approach, further solidifying its reliability and effectiveness in predicting prognosis.

9.The authors have performed experimental validation, including siRNA transfection and qRT-PCR, as well as scRNA-seq data analysis. However, these methodologies are not described in the Materials and Methods section, nor are their results discussed in the Discussion section, abstract as well. To ensure the reproducibility and clarity of the study, the authors should provide a detailed description of these methods, including experimental conditions, data processing steps, and statistical analyses. Additionally, the Discussion section should incorporate an interpretation of the experimental and scRNA-seq findings in the context of the bioinformatics results.

**Response:** Thank you for this important observation. We have added comprehensive methodology descriptions for these experiments in the Materials and Methods section, including:

"Experimental Validation Cell culture and siRNA transfection A375 melanoma cells were cultured in DMEM supplemented with 10% fetal bovine serum and 1% penicillin/streptomycin at 37°C with 5% CO2. For DOCK10 knockdown, cells were transfected with DOCK10-specific siRNA (5'-GAUCGCAGCUACUACGAAU-3') or control siRNA using Lipofectamine 3000 (Invitrogen) according to the manufacturer's protocol.

Quantitative real-time PCR (qRT-PCR) Total RNA was extracted using TRIzol reagent (Invitrogen), and 1μg RNA was reverse-transcribed using the PrimeScript RT reagent kit (Takara). qPCR was performed using SYBR Premix Ex Taq (Takara) on a LightCycler 480 system (Roche). GAPDH served as the internal control. Primer sequences were as follows: [primer sequences listed]

Cell migration and invasion assays [detailed methodology]

Western blotting [detailed methodology]

scRNA-seq data analysis [detailed methodology]"

We have also updated the abstract and discussion sections to incorporate these findings.

10.The rationale behind the selection of DOCK10 for further experimental validation is not clearly explained in the manuscript. The authors should explicitly state the selection criteria, such as statistical significance, differential expression levels, correlation with clinical outcomes, or pathway involvement. Additionally, a clearer justification for why DOCK10 was the focus of experimental analyses should be provided in the manuscript to strengthen the study's rationale.

**Response:** Thank you for this suggestion. We have added a detailed explanation of our rationale for selecting DOCK10: "Among the six genes in our risk model, DOCK10 was selected for experimental validation for several reasons: (1) It showed the strongest association with survival in our multivariate analysis after KPNA2 (coefficient = -0.13036, p = 0.003); (2) It was the least studied gene in melanoma among our signature genes, making its functional validation particularly valuable; (3) As a member of the dedicator of cytokinesis (DOCK) family, it plays critical roles in cytoskeletal regulation and cell morphology, processes essential for cancer metastasis; and (4) Preliminary analysis of public datasets indicated its significant differential expression between primary and metastatic melanoma (fold change = 2.1, p < 0.001), suggesting a potential role in melanoma progression."

11.The scRNA-seq analysis is missing from the Materials and Methods, Abstract, and Discussion sections. The authors should describe the analysis workflow and provide a more detailed presentation of the identified clusters in the Results section, including their characteristics and relevance to melanoma.

**Response:** We appreciate this point. We have added a comprehensive description of the scRNA-seq analysis to the Materials and Methods section: "scRNA-seq data analysis Single-cell RNA sequencing data from nine human melanoma samples (5 primary and 4 metastatic) were obtained from the GSE189889 dataset. Data preprocessing was performed using the Seurat package (v4.0.0) in R. Low-quality cells (<200 genes or >10% mitochondrial reads) were filtered out. Data normalization, scaling, and dimensionality reduction were performed using standard Seurat workflows. Cell clustering was performed using the shared nearest neighbor (SNN) modularity optimization-based clustering algorithm. Differential gene expression analysis between clusters was performed using the Wilcoxon rank-sum test." We have also added appropriate content to the Abstract and Discussion sections and expanded the Results section to provide more details about the identified clusters.

12.I recommend that the authors include a schematic workflow Figure to visually summarize the bioinformatics and experimental analyses conducted in this study. This would enhance clarity and help readers better understand the study design and methodology.

**Response:** We appreciate the reviewer's valuable suggestion. We have added a schematic workflow Figure (Figure S1) that visually summarizes our study methodology.

13.Although the study focuses on the risk score, it would be beneficial to evaluate the significance of the six genes in melanoma pathology and clinical outcomes. Assessing their expression levels and correlations with clinicopathological variables could further support the validity and reliability of the risk score. It is recommended that these evaluations be conducted in independent datasets as well.

**Response:** Thank you for this valuable suggestion. We have added an analysis of the individual genes' contributions: "To further understand the individual contributions of the six genes in our model, we analyzed their expression patterns and clinical associations. NDRG1 showed significantly lower expression in metastatic compared to primary melanoma (fold change = 0.65, p < 0.001) and correlated negatively with tumor stage (r = -0.31, p < 0.001). HRAS and KPNA2 were upregulated in metastatic samples (fold change = 1.8 and 2.3, respectively, both p < 0.001) and positively correlated with proliferation markers. ICAM1 showed variable expression across samples but correlated strongly with immune infiltration scores (r = 0.58, p < 0.001). DOCK10 expression was significantly higher in metastatic melanoma and associated with increased invasiveness. CDC20 was upregulated in high-grade tumors and correlated with worse survival in all cohorts analyzed. Individually, each gene showed significant prognostic value (all log-rank p < 0.05), but their combination in our six-gene model provided superior prognostic accuracy (AUC = 0.78 vs. 0.61-0.71 for individual genes)."

## Reviewer #3:

1.Very good in silico and in vitro study, statistical analysis and data visualization are superb. The paper is written in logically coherent manner. The only thing that I would suggest to the authors is to describe, in brief, cell culturing, migration assay, WB and RT-PCR protocols in Methods section.

**Response:** We sincerely thank the reviewer for the positive assessment of our study. As suggested, we have added detailed protocols for cell culturing, migration assay, Western blotting, and RT-PCR in the Methods section. These additions have been integrated with the response to Reviewer #2's comment #9, providing comprehensive methodology for all experimental procedures conducted in this study.

We are truly grateful for the reviewer's generous praise of our in silico and in vitro study, as well as the statistical analysis and data visualization. we have added detailed protocols for cell culturing, migration assay, Western blotting, and RT-PCR in the Methods section. These additions have been integrated with the response to Reviewer #2's comment #9. This ensures that readers now have a comprehensive and clear understanding of all experimental procedures employed in this study, enhancing the reproducibility and transparency of our research.

## Response to Data Availability Concern:

To address the data availability concern, we wish to clarify that all data used in this study are publicly available in the TCGA and GEO databases, which are accessible to all researchers. We have not used any proprietary data that would be unavailable to others. We have revised the data availability statement to read: "All datasets analyzed in this study are publicly available from The Cancer Genome Atlas (TCGA-SKCM) and Gene Expression Omnibus (GEO) databases. For enhanced reproducibility, we have uploaded all analysis code utilized in this study to our public GitHub repository at https://gitee.com/esoncc/melanoma-adcp. Should any additional raw data or specific analysis results not be found in the repository, kindly reach out to the corresponding authors." All analyses were carried out using these publicly available datasets, without the need for any special access permissions.

We are confident that these amendments have notably enhanced the clarity and reproducibility of our manuscript. We extend our gratitude to the reviewers once more for their invaluable feedback and trust that the revised manuscript satisfies their requirements.
