# Supplementary material for: Exploring the impact of antibody-dependent cellular phagocytosis-related genes on the prognosis of metastatic melanoma
Source: PLoS One. 2025 Oct 9;20(10):e0333916. doi: 10.1371/journal.pone.0333916 (PMC12510546; doi:10.1371/journal.pone.0333916)
Supplement: Attachment — Submitted filename: 7.19 Response to Reviewer v2.docx [file pone.0333916.s007.docx]

## Response to Reviewer #1

**Reviewer #1 Comment:** Thank you for responding to the comments and addressing them. Provided justification and modifications made to the paper looks good.

**Response:** We sincerely appreciate your positive feedback and recognition of our efforts to address your previous comments. Your constructive suggestions have significantly improved the quality of our manuscript. We are grateful for your time and expertise in reviewing our work.

## Response to Reviewer #2

**Reviewer #2 Comment 1:** The manuscript continues to include informal or conversational phrases that are not appropriate for scientific writing. For example, the sentence: "In response to reviewer suggestions, we also performed..." should be revised to maintain a formal academic tone. A thorough review of the manuscript is advised to improve clarity, language consistency, and fluency throughout. Additionally, typographical inconsistencies (e.g., capital "P" in Adj. P-value) should be corrected.

**Response:** We thank the reviewer for this important observation. We have thoroughly reviewed the entire manuscript and removed all informal phrases. Specifically:

1. The phrase "In response to reviewer suggestions, we also performed..." has been revised to: "**An additional analysis using the entire TCGA dataset (n=351) was performed for model development to strengthen the statistical power for coefficient estimation. The model coefficients remained stable with both approaches, confirming the robustness of our six-gene signature**" (page 8, lines 161-164).
2. All instances of capital "P" in p-values have been corrected to lowercase "p" throughout the manuscript. We note that there are a few remaining instances with italicized "P" in Figure 8 legend that should also be changed to "p" for complete consistency.
3. We have conducted a comprehensive language review to ensure formal academic tone and consistency throughout the manuscript.
4. We refined some of the descriptions in Methods (page 11, lines 225-232, page 12, 251-257) and Results(page 11, lines 282-295).

**Reviewer #2 Comment 2:** Despite detailed descriptions of siRNA knockdown, qRT-PCR, and Western blotting in the main text, these efforts are not mentioned in the abstract. Given their importance to the study's conclusions, a brief reference to these experiments in the abstract is necessary to accurately reflect the scope of the work and draw appropriate scientific interest. Furthermore, the discussion section does not critically integrate the experimental findings into the broader implications of the model. A deeper interpretation of the functional significance of DOCK10 knockdown results is recommended.

**Response:** We appreciate this valuable suggestion and have made the following revisions:

1. **Abstract revision** (page 2, lines 25-28): We have added: " **Additionally, comprehensive experimental validation including siRNA knockdown, quantitative real-time PCR, Western blotting, and functional assays was performed to validate the biological role of DOCK10, a key gene in our prognostic model.**"
2. **Abstract Methods section** (page 2, lines 37-40): We added: " **SiRNA-mediated knockdown of DOCK10 in A375 melanoma cells was performed, followed by assessment of proliferation, migration, and invasion capabilities through qRT-PCR, Western blotting, wound healing, and Transwell assays.**"
3. **Abstract Results section** (page 2, lines 54-57): We included: " **Knockdown of DOCK10 in A375 melanoma cells significantly impaired cell proliferation, migration, and invasion, accompanied by reductions in Ki67, ITGB1, and CDH5 expression. These findings confirm DOCK10’s functional contribution to melanoma progression.**"
4. **Abstract Conclusion** (page 2, lines 62-64): We added: "**The functional validation of DOCK10 strengthens the biological relevance of our prognostic model and identifies DOCK10 as a potential therapeutic target for melanoma metastasis.**"
5. **Discussion section enhancement** (page 16, lines 17-27 and page 16-17, lines 1-7): We have integrated the experimental findings more thoroughly by adding discussion of how DOCK10's functional validation supports the computational predictions and strengthens the biological foundation of our prognostic model.

**Reviewer #2 Comment 3:** Although DOCK10 appears to be a central gene of interest, the associated functional validation (siRNA, qPCR, migration/invasion assays, Western blot) feels structurally detached from the core risk model. This disjointedness creates the impression of a parallel sub-study, rather than a fully integrated component of the main prognostic framework. A more explicit narrative link between the DOCK10 analyses and the risk score model would improve cohesion and strengthen the manuscript's scientific integrity. I would kindly recommend that the authors re-evaluate the manuscript holistically, ideally from the perspective of a reader unfamiliar with the study, to ensure that all sections are conceptually connected and narratively coherent.

**Response:** We greatly appreciate this insightful comment. We have restructured the narrative to better integrate DOCK10 validation with the prognostic model throughout the manuscript:

1. **Introduction** (page 5, lines 105-108): We added: "**To strengthen the biological relevance of our findings, we performed comprehensive functional validation of DOCK10, a key gene in our prognostic model, demonstrating its critical role in melanoma cell proliferation, migration, and invasion.**"
2. **Results section** (page 15, lines 320-327): When introducing DOCK10 selection, we now state: "**Among the six signature genes, DOCK10 was selected for in-depth functional validation. DOCK10, a member of the dedicator of cytokinesis (DOCK) family that orchestrates cytoskeletal remodeling and cell morphology which are key steps in metastatic dissemination with significant prognostic value (multivariate Cox coefficient = –0.13036; p = 0.003). Notably, DOCK10 expression was markedly higher in metastatic versus primary melanoma (fold change = 2.10; p < 0.001), underscoring its putative role in melanoma progression and justifying further experimental characterization.**"
3. **Results - Validation of Risk Genes** (page 16, lines 437-448): We added: " **Although we have established a prognostic model for metastatic melanoma based on six ARGs (NDRG1, HRAS, KPNA2, ICAM1, DOCK10, and CDC20), to determine which gene plays a key role in melanoma metastasis, we further integrated single-cell transcriptomic data from primary and metastatic melanomas. Through filtering, dimensionality reduction clustering, and cell annotation, we identified eight cell subpopulations (Figure 8A). Notably, melanoma cells from metastatic tissues showed significantly higher DOCK10 expression compared to primary melanoma tissues (Figure 8B, C). Furthermore, we divided melanoma cells into high and low DOCK10 expression groups (Figure 8E). Through differential gene enrichment analysis using gene ontology (GO) (Figure 8D), we found that the high DOCK10 expression group was highly enriched in cadherin binding and focal adhesion pathways, which are related to cell adhesion.**" In addition, we have added Figure S3, which presents the expression levels of NDRG1, ICAM1, KPNA2, HRAS, and CDC20 in different cell clusters in metastatic and primary melanoma.
4. **Results - Experimental validation**: We emphasized: " **To validate DOCK10's functional contribution to melanoma progression, we performed siRNA-mediated knockdown in A375 melanoma cells.**" (page 21, lines 449-450) **Ki67 serves as a well-established marker of cell proliferation. Compared to control siRNA, transfection with DOCK10 siRNA markedly decreased Ki67 expression in A375 cells, suggesting a potential suppression of cell proliferation (Figure 8H). Invasion and migration are crucial processes in tumor cell motility. The invasive potential of A375 cells was evaluated using the Transwell assay. As depicted in Figure 8I, the knockdown of DOCK10 significantly inhibited the invasiveness of A375 cells. To further assess cell migration, wound healing assays were conducted. Compared to control siRNA, silencing DOCK10 with siRNA notably impaired the migration of A375 cells (Figure 8J). The mRNA levels of CDH5, a protein implicated in cell migration and invasion, were reduced in A375 cells upon DOCK10 knockdown, as evidenced by qRT-PCR (Figure 8K). Western blot analysis was subsequently performed to measure the expression of proteins associated with cell proliferation, migration, and invasion. The findings revealed that DOCK10 siRNA markedly decreased the protein expression of Ki67, ITGB1, and CDH5 in A375 cells relative to control siRNA (Figure 8L).** (page 21, lines 455-469)
5. **Results - Conclusion of validation** (page 22, lines 469-473): We added: " **These comprehensive functional studies demonstrate that DOCK10 knockdown significantly impairs multiple hallmarks of melanoma progression, including proliferation, migration, and invasion. These evidence not only validate DOCK10 as a driver of metastatic behavior but also reinforce its candidacy as a therapeutic target within our ARG‐based prognostic model.**"
6. **Advantages and Limitations**: We better integrated DOCK10's role by adding: " **The integration of computational analysis with experimental validation, particularly the functional characterization of DOCK10, provides mechanistic insights that strengthen the biological foundation of our prognostic model.** " (page 27, lines 578-581) " **While we provided functional validation for DOCK10, comprehensive experimental validation of all six genes in the model would further strengthen our findings. Future studies should focus on validating the remaining genes and elucidating their collective contribution to melanoma progression. Although we have established a prognostic model for metastatic melanoma based on six ARGs (NDRG1, HRAS, KPNA2, ICAM1, DOCK10, and CDC20), in order to identify which gene plays a key role in melanoma metastasis, we further integrated single-cell transcriptomic data from primary melanoma and metastatic melanoma.Among these six genes, we found that DOCK10 is highly expressed in metastatic melanoma compared to primary melanoma. Therefore, DOCK10 may be an important driving factor in melanoma metastasis. Furthermore, we divided the melanoma cells into two groups based on the expression levels of DOCK10: high and low expression groups. Through differential gene enrichment analysis, we found that melanoma cells with high DOCK10 expression were highly enriched in the cadherin binding and focal adhesion pathways.Therefore, we hypothesize that DOCK10 plays an important role in the adhesion to endothelial cells during melanoma metastasis. We further knocked down DOCK10 in A375 cells using siRNA, and the results showed a significant decrease in the adhesion of A375 cells to endothelial cells. Additionally, Western blot experiments confirmed that the expression of adhesion-related proteins, ITGB1 and CDH5, was significantly reduced after DOCK10 knockdown in A375 cells. Moreover, the knockdown of DOCK10 resulted in a marked decrease in the proliferation, migration, and invasion abilities of the cells.Therefore, our experimental validation definitively demonstrated that DOCK10 knockdown impairs melanoma cell proliferation, migration, and invasion, confirming its functional significance in melanoma progression. This direct evidence of DOCK10's role in melanoma metastasis not only validates our computational predictions but also strengthens the biological foundation of our ARG-based prognostic model. Our scRNA-seq analysis provided additional insights into DOCK10's role by revealing its elevated expression in metastatic melanoma cells compared to primary melanoma cells at single-cell resolution. This cell-type specific expression pattern, combined with our functional validation showing DOCK10's critical role in melanoma cell behavior, establishes a mechanistic link between DOCK10 expression and melanoma progression. The convergence of computational prediction, single-cell expression analysis, and functional validation exemplifies the power of integrating multi-level evidence to understand prognostic gene signatures.** " (page 27, lines 590-624)
7. **Conclusion**: We better concluded DOCK10's role by adding: **The experimental validation of DOCK10 not only confirms its role as a key driver of melanoma metastasis but also validates our computational approach for identifying clinically relevant ADCP-related genes. This integration of bioinformatics prediction with functional validation establishes a paradigm for developing biologically grounded prognostic models that can guide both patient stratification and therapeutic target discovery in metastatic melanoma.** (page 30, lines 630-636)

**Reviewer #2 Comment 4:** The explanation provided for omitting the heatmap of the 887 common DEGs due to “space limitations” is not fully convincing.

**Response:** We greatly appreciate your constructive feedback. We recognize the importance of the heatmap for the scientific rigor of the article. To address this concern, we have included a heatmap of the 887 common DEGs in Figure S2 in the Supplementary Materials. In addition, the expression levels of each gene have been summarized in Supplementary Table 1.

**Reviewer #2 Comment 5:** A schematic summary of the study workflow remains missing. Given the complexity of the multi-step bioinformatics and validation pipeline, a figure summarizing the analytical flow would greatly aid comprehension.

**Response:** We thank the reviewer for this suggestion. We have addressed this by:

1. **Adding a workflow figure** as Figure S1 in the Supplementary Materials.
2. **Revising the Results section** (page 14, lines 282-283) to reference this figure: " **In this study, we implemented a multi‐step analytical pipeline that combined large‐scale bioinformatic screening with targeted experimental validation (Figure S1).**"
3. **Adding Figure S1 legend** (page 50, Supplementary Figure Legends): "**Figure S1. Workflow diagram of the study. The flowchart illustrates the systematic approach used in this research, including: (1) Data acquisition from TCGA and GEO databases; (2) Identification of differentially expressed genes between primary and metastatic melanoma; (3) Intersection with ADCP-related genes; (4) Feature selection using LASSO regression; (5) Construction of the six-gene prognostic model; (6) Validation in multiple cohorts; (7) Functional analyses including pathway enrichment, immune cell infiltration, and drug sensitivity prediction; (8) Experimental validation of DOCK10 through siRNA knockdown and functional assays.**"

## Response to Reviewer #3

**Reviewer #3 Comment:** Authors have described the protocols of in vitro experiments in Methods section of the manuscript and that makes all of my comments addressed.

**Response:** We sincerely thank you for your positive feedback and for acknowledging that we have adequately addressed your concerns regarding the experimental protocols. Your previous suggestions to include detailed descriptions of the in vitro experiments have significantly improved the completeness and reproducibility of our work. We appreciate your time and effort in reviewing our manuscript.
