# Supplementary material for: Exploring the impact of antibody-dependent cellular phagocytosis-related genes on the prognosis of metastatic melanoma
Source: PLoS One. 2025 Oct 9;20(10):e0333916. doi: 10.1371/journal.pone.0333916 (PMC12510546; doi:10.1371/journal.pone.0333916)
Supplement: Attachment — Submitted filename: 9.12 Response to Reviewers .docx [file pone.0333916.s008.docx]

## Response to Reviewer #2

**Reviewer #2 Comment 1:**

The abstract exceeds optimal length for PLOS One. Authors are recommended to suggest improving conciseness, grammar, and flow for clarity and readability. For instance,

Phrases such as “a nomogram was industrialized” (Line 36) should be revised (e.g., “constructed” or “developed”).

Line 51: “which is allied with poorer prognosis” should be rephrased as “associated with” for clarity.

Line 61: “prominence its potential utility” is unclear; consider revising to “highlighting its potential utility”.

Several molecular techniques (qRT-PCR, Western blotting, functional assays) are mentioned multiple times, causing repetition.

The background should focus on the scientific rationale and hypothesis, not techniques. A well-edited and concise abstract will better reflect the strength of the study.

**Response:**

We sincerely thank the reviewer for the constructive comments and valuable suggestions that have greatly helped us improve the quality of our manuscript. The revisions made in response to these suggestions are detailed below:

1: We have condensed the abstract by removing redundant phrases and unnecessary details while retaining the core scientific findings and methods. The revised abstract is now under 300 words and more concise. We focused on the most relevant results and the significance of our findings for clinical decision-making in metastatic melanoma.

2: We have replaced "industrialized" with "developed" (Line 27) to align with standard academic writing, as suggested by the reviewer.

3: We have revised this phrase to "associated with poorer prognosis," (Line 37) which is more precise and academically appropriate.

4: The phrase has been revised to "highlighting its potential utility," (Line 41) improving clarity and flow, as recommended.

5: We have streamlined the mention of these techniques. The details are now included in the methods section only once, rather than repeating throughout the abstract. This reduces redundancy and keeps the focus on the key findings.

6: We have revised the background to focus more on the scientific rationale and research hypothesis, removing excess detail about the experimental techniques. This makes the abstract more focused on the core scientific questions and insights of the study.

Additional Changes Made:

Overall Clarity and Readability: We have revised the abstract to improve its flow and ensure it is easier to read and understand. Unnecessary technical jargon was reduced, and the overall language was simplified for better accessibility to a broad audience.

Thank you for your helpful suggestions! We believe the changes improve the clarity and impact of our abstract and manuscript.

**Reviewer #2 Comment 2:**

The inclusion of heatmaps for the 887 shared DEGs across GSE7553 and GSE46517 is appreciated. However, the current visual presentation could be further improved:

The upper sample-type color bar is redundant, given that sample types are already represented in the lower legend. Its removal would streamline the figure.

The scale bar lacks appropriate labeling, and the normalization method is not indicated. Please clarify this either on the figure or in the legend.

Importantly, the heatmaps lack dendrograms, making it difficult to assess whether hierarchical clustering was performed on genes and/or samples. Including clustering branches would enhance interpretability and biological insight.

We recommend these final adjustments to enhance the clarity and rigor of the manuscript before acceptance.

**Response:**

Thank you for the helpful suggestions regarding the heatmap presentation. We have made the following revisions:

1: Sample-type color bar: We removed the upper “sample-type” label as it duplicated the information already shown in the lower legend.

2: Scale bar and normalization: The heatmaps were generated with the R package pheatmap using scale = "row". This performs z-score normalization (value minus the row mean, divided by the row standard deviation). We have added this information next to the scale bar and in the figure legend.

3: Dendrograms: We now display dendrograms for genes in the revised figure, indicating hierarchical clustering performed after the row-wise z-score transformation. (Clustering in pheatmap was run with default settings.) The sample order is kept as in the experimental design and is stated in the legend.

We believe these changes improve clarity and interpretability, and we appreciate the reviewer’s constructive feedback.
